# Phase of Shear Vibrations within Cochlear Partition Leads to Activation of the Cochlear Amplifier

**DOI:** 10.1371/journal.pone.0085969

**Published:** 2014-02-14

**Authors:** Jessica S. Lamb, Richard S. Chadwick

**Affiliations:** 1 Section on Auditory Mechanics, National Institute on Deafness and Other Communication Disorders, Bethesda, Maryland, United States of America; University of Salamanca- Institute for Neuroscience of Castille and Leon and Medical School, Spain

## Abstract

Since Georg von Bekesy laid out the place theory of the hearing, researchers have been working to understand the remarkable properties of mammalian hearing. Because access to the cochlea is restricted in live animals, and important aspects of hearing are destroyed in dead ones, models play a key role in interpreting local measurements. Wentzel-Kramers-Brillouin (WKB) models are attractive because they are analytically tractable, appropriate to the oblong geometry of the cochlea, and can predict wave behavior over a large span of the cochlea. Interest in the role the tectorial membrane (TM) plays in cochlear tuning led us to develop models that directly interface the TM with the cochlear fluid. In this work we add an angled shear between the TM and reticular lamina (RL), which serves as an input to a nonlinear active force. This feature plus a novel combination of previous work gives us a model with TM-fluid interaction, TM-RL shear, a nonlinear active force and a second wave mode. The behavior we get leads to the conclusion the phase between the shear and basilar membrane (BM) vibration is critical for amplification. We show there is a transition in this phase that occurs at a frequency below the cutoff, which is strongly influenced by TM stiffness. We describe this mechanism of sharpened BM velocity profile, which demonstrates the importance of the TM in overall cochlear tuning and offers an explanation for the response characteristics of the Tectb mutant mouse.

## Introduction

Mammals transduce sound waves to neuronal signals within the fluid-filled cochlea via traveling waves in the organ of Corti (OC). This mechanical process is responsible for much of the extraordinary range and sensitivity that is characteristic of mammalian hearing, yet underlying mechanisms of nonlinear perception that enable the most interesting aspects of hearing, are still being determined [1], [27]. Investigators probe cochlear mechanics through a wide variety of methods, including velocimetry of the basilar membrane (BM) [3], multidirectional measurements of tectorial membrane (TM) motions [4], and recently measurements of the sensory epithelia, the reticular lamina (RL) [5]. However, these methods all offer glimpses of limited sections of the cochlea, so a well developed traveling wave model can help interpret the results. Sound manifests in the inner ear as a transverse traveling wave on the tissue of the OC. Mechanical frequency separation occurs via gradients in the mass and viscoelastic properties of the tissue that cause the position of peak vibration to depend on frequency. The precise mechanical structure of the mammalian cochlea has inspired many attempts to elucidate the roles of the different substructures through modeling. A useful way to examine the whole traveling wave is with a model based on the Wentzel-Kramers-Brillouin (WKB) approximation, a subset of perturbation theory ideal for analyzing waves propagating in slowly changing media. Originally, the approach was developed by using a simple, flexed basilar membrane (BM) to partition the cochlea fluid [6]. While outer hair cells (OHC) and their stereocilia are accepted as the primary source of nonlinearity, researchers continue to investigate the role other structural features have on the tuning curve. The tectorial membrane (TM) has been suggested as a possible source of secondary filtering or resonance [7], [8]. Previously, we increased the complexity of the cochlea WKB model by developing it with a modified “sandwich” cross-section [9], allowing the TM, which has a fluid-facing surface, to vibrate as a second degree of freedom and found that such a system can produce a second propagating wave [10]. However, this simple system did not include shear between the TM and RL, which must be an input for any amplification mechanisms involving OHCs [11]. Here, we expand that model and present an active dual wave model with three self-equilibrating degrees of freedom by including a shear motion at an angle set in the model. From this we see that relative phase of vibrating structures strongly affects active force output, enabling us to offer a novel explanation for observations of the Tectb mutant mouse [12]. We also consider longitudinal coupling within the tissue and the affect RL angle has on activity.

## Methods

This model builds on results in [10], the aforementioned model where differential motion of the TM generates a second mode, and [13], which added a nonlinear active force onto a WKB-based model as a perturbation. The complete derivation of this model comprising aspects of both of these papers as well as the strategies discussed below, can be found in File S1. Here we focus on explaining the novel aspects of this work: the inclusion of the shear angle 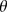, between the RL and TM, and the active force perturbation worked out in the two wave system.

### Lumped Parameter Model with Hair Bundle Shear

**Figure 1:**
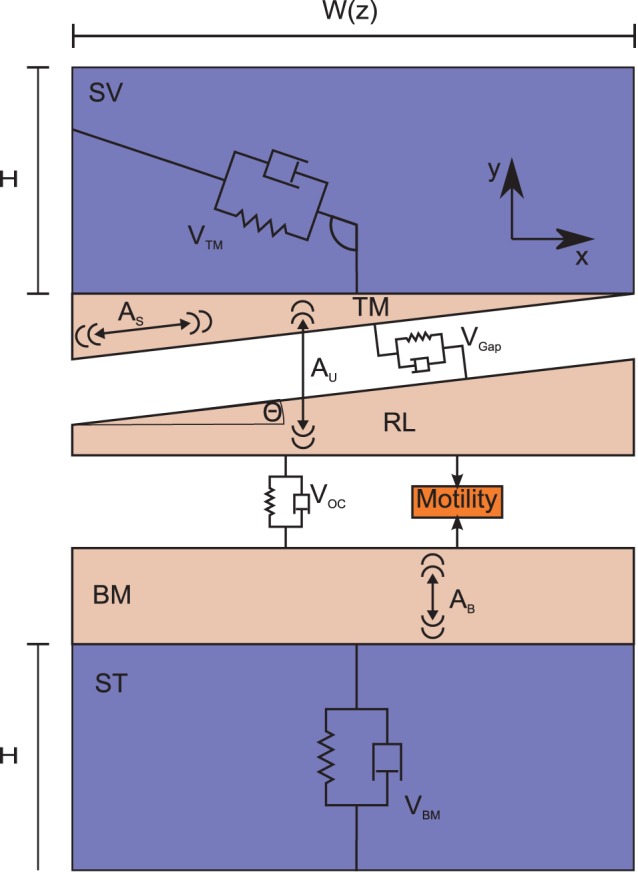
Illustration (not to scale) of the lumped-parameter cross section of the cochlea. This figure illustrates the scala height (H), OC width (W(z)), tissue masses (M), and viscoelastic elements (V). The direction of V_TM_ is drawn at an arbitrary angle - the force always acts in a direction that restores the TM to its resting position.

The cochlea is modeled as a long, fluid-filled duct partitioned into two compartments, the scala vestibule (SV) and scala tympani (ST), roughly corresponding to the physiology. The lumped masses, 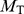, 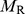 and 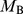 (subscripts indicating TM, RL and BM respectively) are proportional to cochlear width, *W(z)*, where *z* is the long dimension. This and other dimensions are based on observations for the mouse [14]. There are few measurements of viscoelastic properties in mice, and those that exist are difficult to interpret as single spring constants, so stiffness coefficients are based on the observed frequency range of a mouse, and increase from base to apex by a single exponential gradient. The ratio of damping to stiffness for the TM was determine from observations in [15] (rough calculations based on these moduli and the dimensions of the mouse TM suggest the stiffness values we use are of the correct order of magnitude.) Damping was otherwise kept small, increasing by an exponential coefficient that is half that for the stiffness (Tables S1 and S2 in File S1). We balance the internal forces of the OC with the fluid by considering the lumped-parameter cross section illustrated in Fig. 1, which defines most of the model viscoelastic and dimensional constants.

Some simplifying approximations specific to this model design are discussed as follows: by introducing the shear between the TM and RL we also introduce the possibility of two-dimensional motion the TM. Thin-film viscous adhesion will keep the gap between the TM and reticular lamina (RL) constant. The direction of the viscoelastic force due to the TM (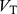) varies such that it always works to restore the mass to its zero position. These approximations, and those in our previous work [19] lead to a tractable analytical model.

The partition equation relates the forces from the internal viscoelastic properties and displacements of the cochlear partition (CP) to the fluid pressure. These displacements are expressed using vector notation (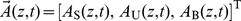) where the subscripts denote the shear, upper, and basilar components respectively.
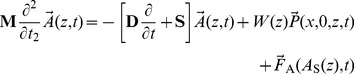
(1)Here **M**, **D**, and **S** are slowly varying matrices describing respectively the internal mass, damping, and stiffness per unit length of the OC (Fig. 1 gives the subscript notation for each structure) and 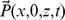 is a vector of fluid pressure at the partition (located at 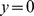). 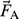 represents the active force, discussed below. Because all viscoelastic elements have mass and damping terms in parallel, the matrices **D** and **S** have the same form, and we represent them both with **V**. The cross section matrices and form of the pressure vector can be derived by balancing forces due to the elements of the CP and the forcing fluid pressure. The matrices are



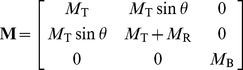
(2)

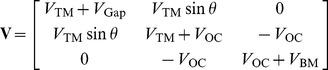
(3)


To derive an analytical expression relating 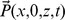 to 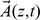, we recognize that the problem is one of multiple scales, where the cross section and wavelength dimensions are much shorter than the overall length of the cochlea. We thus use a WKB method to expand both quantities around the small number 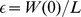, where 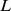 is the length of the cochlea, the ratio of the short to the long scales. We proceed to solve Laplace’s equation in both fluid chambers, using 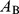 or the total displacement of the TM, 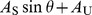, to describe the partition boundaries [10]. Combining this with the force balance in the cross section, the pressure term on the RHS of Eq. (1) becomes
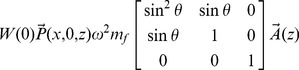
(4)where 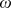 is the driving frequency, 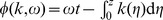 is the accumulated phase and 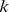 is the wavenumber. The matrix is brackets will be shown as 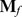 and
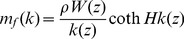
(5)with 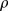 being the fluid density. All of the solutions will have the form 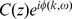, where 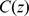 is 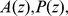
… as appropriate.

### Active Force

Somatic electromotility in OHCs [16] implies the possibility of force applied between the RL and BM *in vivo*. The active force in this model is developed with this force profile and a standard sigmoid function, without further specifying the origin of the dominant nonlinearity. A Fourier coefficient is generated for the fundamental frequency and harmonics are neglected in the solution. The active force term can then be written as.
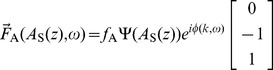
(6)where 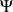 is the Fourier coefficient defined as
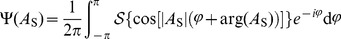
(7)and 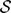 is the sigmoid function



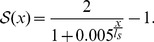
(8)The range of the function is scaled by the input saturation length, defined by the inverse of the sigmoid function 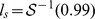. The output is normalized with the maximum force given explicitly by 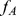. To use the perturbation method in [13] we recognize that 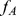 is much smaller than the scale of the inertial forces, i.e. 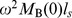. The ratio of the two gives a small number, 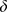, about which we expand 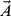 and *k*. We also introduce the subscript notation 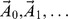 to indicate the order the displacement after treated with the perturbation expansion. This gives us the homogeneous and perturbed equations
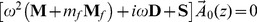
(9)




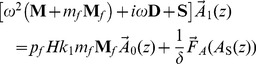
(10)where the enumerated subscripts indicate expansion orders and 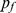 is a coefficient dependent on 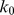 that arises through the small number expansion and is expressed in File S1.

### Solution

Eq. (9) represents the passive problem, which we solve as in [10] and then use the results to determine the active contribution. Briefly, this is a generalized eigenvalue problem for 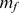 that can be solved computationally, using the WKB transport equation and the boundary conditions at the base to scale the eigenvectors. Although this model has three coupled equations, because 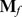 is singular there are only two eigenmodes. The passive response of one mode is strongly influenced by the properties of the TM, and has larger amplitude on that structure, while the same can be said of the other and the BM, motivating the use of ^
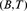
^ to denote each mode. Given this, the active system can be described via the expansions
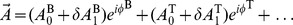
(11)




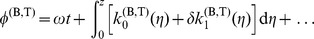
(12)


Once the passive model is solved, we can use the resulting 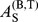 to calculate 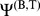 for each eigenmode. Eq. (10) is only solvable if the RHS is orthogonal to the eigenvector of the homogeneous equation, and from this solvability condition we can derive the expression for 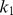, the active wavenumber correction
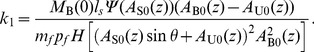
(13)


The LHS of Eq. (10) requires choosing an eigenmode to set 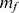. Obviously the homogeneous solution will be the eigenvector for this mode; less obviously the particular solution will be the eigenvector of the other mode, which we need to scale. By assuming that the contribution from the homogeneous solution is entirely accounted for in the passive problem and setting it to 0, we can scale 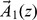 by solving Eq. (10). This involved expression is given in File S1.

### Additional Terms

We wished to consider the effect of longitudinal coupling in the TM, both to test the effect of a longitudinal coupling source besides the fluid on our model. We developed a term to be added to a two degree of freedom model, without any shear motion. 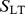 represents longitudinal spring constants. A “waves on a string” problem typically involves taking two derivatives in the direction of wave propagation, 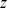 in our model. However, in the WKB approximation we have employed, these derivatives are replaced by the wavenumber, we add the term
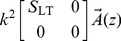
(14)to the passive equation of motion. The active expansion derived in [13] cannot be worked with this term in place, but the passive model can still provide some insight.

## Results and Discussion

### Passive Model Comparison

Table 1 shows the zeroth order wavenumbers for the original passive model, that with added longitudinal coupling in the TM, and that with shear. The wavenumbers with large real and small imaginary components indicate propagating modes, and there are clearly two for each model. This is significant because neither the extra degree of freedom introduced by shearing, nor the additional longitudinal coupling added propagating waves. Previous works have dealt with evanescent wave modes, showing they make a perceptible contribution near the frequency cutoff [17], [18], or than they emerge with added components such as longitudinal coupling [19] and fluid chambers [20]. However, to our knowledge only another sandwich model [9] suggested an additional propagating mode is possible. We conclude the fluid-partition interfaces carry propagating modes like surface waves. This analysis demonstrates that only independent motions of the fluid-facing surfaces of the CP, the TM and BM create propagating waves. Since the CP is a viscoelastic structure, such independent motions could certainly arise *in vivo*, making the second wave an important consideration.

**Table 1:** Wavenumbers with imaginary parts ordered from least to most negative for three models.

Independent TM only	Longitudinal TM coupling	Shearing between TM and RL
69.00–00.39*i*	70.65–0.30*i*	68.96–0.39*i*
91.37–01.64*i*	27.04–25.26*i*	91.64–1.66*i*
0.32–888.52*i*	0.05–892.11*i*	0.32–889.46*i*
0.06–892.37*i*	0.02–1792.46*i*	0.06–892.37*i*

Values are presented in units of mm^−1^ and calculated at 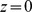 where the waves are longest and least likely to be evanescent.

Also of note is the extreme similarity of wavenumbers between the system with and without shear. Adding this component lets us extract more information about the system, but the waves are essentially the same. Thus conclusions about the two degree of freedom system [10], including those regarding relative phases of the two modes and mode conversion, are valid as we increase model complexity.

### Varying TM Stiffness

**Figure 2:**
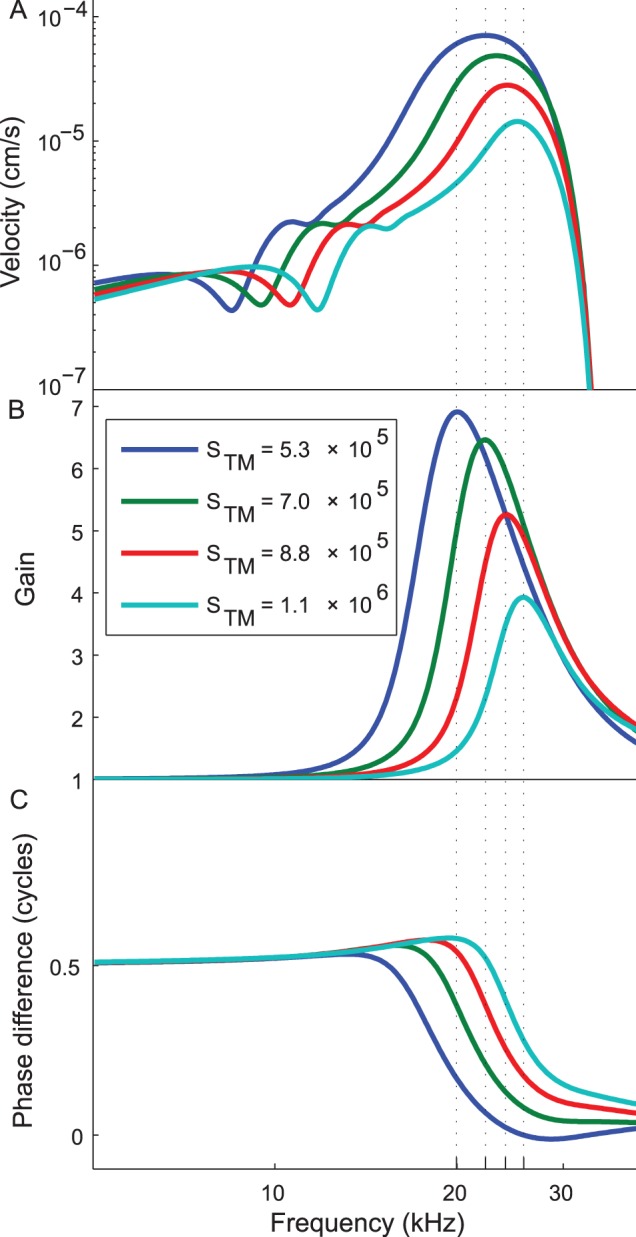
Demonstration of the frequency response on TM stiffness. Vibrations calculated at 1.4/mm and stiffness are given per length in units of N/mm^2^. Fig 2a: As stiffness increases, velocity peaks decrease and narrow. Fig 2b: Gain (in the BM mode) calculated as the quotient of the active displacement over the passive displacement peaks at progressively higher frequencies with increasing stiffness. Vertical lines correspond to maximum gains. Fig 2c: The phase difference between the shear vibrations and the BM vibrations in the BM mode undergoes a dramatic shift from out-of-phase to in-phase at a frequency that is strongly dependent on TM stiffness. This shift seems to correspond well with best gain the beginning of the active force peak.

The most significant result we found was that by increasing the stiffness of TM, the selectivity (as defined by the quality factor) of the BM increased, but the sensitivity (as defined by the maximum velocity) decreased, as shown in Table 2. Fig. 2 shows how the frequency responses of BM velocity and other quantities change with the TM stiffness. To maximize the impact of the active force we use an input SPL of 20 dB (referenced to 20 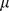
Pa, as is standard, and without modeling the middle ear transfer function, which likely adds some further gain [21].) We can see the decrease in quality factor corresponds to a shift in the region of significant gain. After testing the frequency of this transition against many model parameters, such as BM stiffness, OC stiffness, and TM damping (see File S1), we find it is strongly dependent on the TM stiffness and fairly insensitive to any other parameter examined.

**Table 2:** Quality factors and sensitivities for different TM stiffnesses.

TM stiffness (N/mm^2^)	Q	Maximum Velocity (mm/s)
0.05	2.4	7.2×10^−4^
0.07	2.8	5.0×10^−4^
0.09	3.4	2.9×10^−4^
0.11	3.7	1.5×10^−4^

All values calculated at 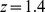 mm.

The wavelength correction defined in Eq. (13) is responsible for most of the active force gain. Examining the equation analytically and looking at predictions for the frequency dependent behavior of the quantities on which 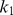 depends do not reveal any dominating transitions that account for the sudden rise in gain, although several of them exhibit small bumps around the start of the gain. The model does predict a strong transition in the phase difference between the shearing motion and the basilar membrane vibration. Although the formula for 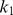 does not explicitly involve this term, it is quite reasonable to expect the amplifier needs to work at a specific phase to the BM vibration to be effective. Indeed, Dong and Olson recently observed a transition the phase difference between the extracellular voltage of the *scala vestibuli*, which is related to the active force, and the fluid pressure near the BM, which indicates its motion [22]. They also suggest this phase shift optimizes the timing of the cochlear amplifier, and if one considers the phase of the active force should mimic the phase of the input shear, their result supports our prediction of a phase transition.

We can think of this transition as restricting the frequency range of the active region, somewhat akin to a high-pass filter. The abrupt loss of passive amplitude at the cutoff frequency restricts on the high-frequency end, together making a narrow band region of gain. Shifting the frequency of the “high-pass filter”, by changing the TM stiffness, but maintaining the frequency cutoff alters the Q of the frequency response. For stiffness values that allow the filtering effects to overlap, the frequency of maximum vibration is impacted by the gain-reducing qualities and is diminished, lowering sensitivity, trading sensitivity for selectivity. The high-frequency end of the band can be altered by varying BM stiffness, giving the same sensitivity-selectivity trade off (see File S1) but the frequency of the phase transition remains constant.

This result is particularly interesting given that velocity threshold measurements on the BM of the high-frequency (basal) region of the Tectb mutant mouse displays increased selectivity and somewhat decreased sensitivity compared to the wildtype [12]. The mutated protein is found extensively in the TM of this animal and the membrane, which at the base lacks an organized striated-sheet matrix and Hensen’s stripe, and is further disrupted at the apex. This TM phenotype and experiments [23] have led researchers to attribute the altered tuning curve to a decrease in longitudinal coupling associated with the loss of the striated-sheet matrix. It is suggested that OHCs on the periphery of the frequency place will be not be engaged in the mutant, leading to a decrease in overall amplification as well as finer tuning [24]. Modeling bears this out as a possible cause of fine tuning, but does not demonstrate decreased sensitivity [25]. Our result offers an alternate explanation, which can achieve the same phenomena based solely on changes in local resonance properties of the TM, without implicating longitudinal coupling.

As part of this interpretation, we must consider how the changes observed in the TM of the Tectb mutant may manifest themselves as the increase in stiffness required by our model. Our lumped stiffness parameter simultaneously represents TM attachment, elastic modulus, and any bending effects which are left out of our model. For modeling purposes we assumed a fairly homogeneous cross section, but the actual cochlea is quite complex, and any inhomogeneity due to fluid pressure, geometry or differential force from the cells versus the numerous fluid spaces in the OC may make the bending characteristics quite important. In this case, a loosely coupled system might in effect be stiffer by requiring a greater fluid pressure at some local point to produce the same amount of force transmitted at another local point (such as directly over the hair cells). Thus the observed disorganization of the TM structure that led other researchers to think about a decrease in longitudinal coupling might increase the lumped parameter spring constant.

### Phase of the Cochlear Amplifier

To amplify BM vibrations, OHC electromotility must be properly timed in the vibration cycle [26]. Nilsen and Russel [27] discuss this and conclude amplification occurs at the maximum velocity of the OHC cycle, which occurs a quarter cycle after the maximum displacement. Chadwick [13] found a factor of *i* was needed for correct timing in his model. Examining Fig. 2, reveals the maximum gain in this model occurs when the RL displacement is nearly a quarter cycle different from the BM, corresponding to these experimental and model results.

**Figure 3:**
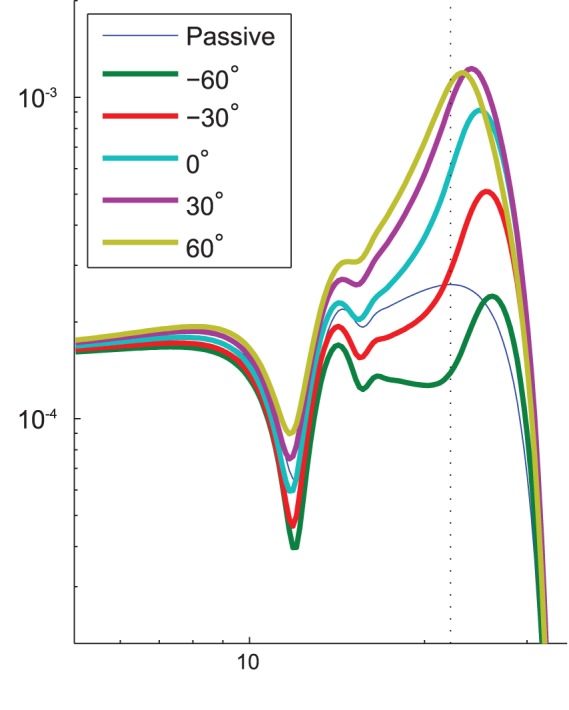
Illustration of the shift in best frequency caused by a shift in the phase of the IO function. The vertical dotted line indicates the best frequency of the passive response.

In general, we assumed the phase of action of the cochlear amplifier was zero. That is, a positive shear of OHC stereocilia causes a contraction at the same point in the phase cycle without any sort of delay [28]. However, since *in vivo* factors including local damping from surrounding tissues and slow electrical response times may introduce delay, we investigated altering the phase of the input-output relation. The maximum velocity in these scenarios, shown in Fig. 3 is smaller, but interestingly the best frequency is also shifted. Even with no phase shift, our model shows peak activity at higher frequency than the passive velocity maximum, consistent with experiments that show the observed the best frequency shifts for very high input sound pressure levels (SPL) [29]. This suggests that phase differences in the amplifier could affect tuning as well as sensitivity.

### Reticular Lamina Angle

**Figure 4:**
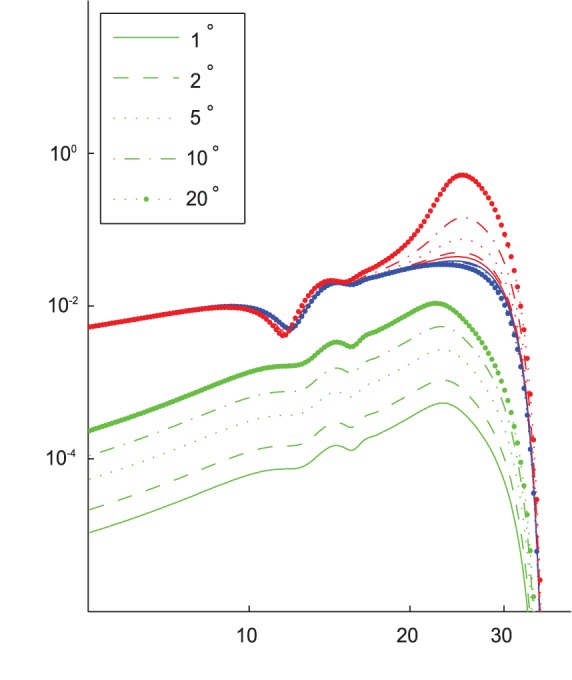
Dependence of vibration on RL angle. Green lines indicate passive shear velocity, blue passive BM velocity, and red active BM velocity.

By introducing the shear angle into the model, we gained a means to examine the role of this quantity which is known to grow from base to apex [14], [30]. While the angle has little effect on the passive BM vibration, a greater angle leads to much greater amplification as shown in Fig. 4. This is due to the larger shearing motion on the stereocilia, which is the input to the active force. Eq. (13) shows the wavenumber correction is directly proportional to this number. The larger angle couples more of the fluid force directed perpendicular to the partition into the shear direction, giving rise to a larger active force. Since this angle increases from base to apex, it may help aid amplification of low frequencies that must be carried far into the cochlea. This echoes work that suggests low frequency sound perception is enhanced at the apex by mechanics that selectively promote shear in this region [31].

## Conclusions

Our model offers an intriguing possibility to explain the simultaneous increase in selectivity with loss of sensitivity of the Tectb mutant - a band pass filtering mechanism, with the low corner being determined by the TM-BM interaction. This idea lends further support to previous studies that suggest the properties of the TM are important to overall cochlear tuning [8], [32], [33] and specifically echoes work that suggests the TM-RL movement can act as a filtering mechanism [7], [11]. This work provides an in depth description of the mechanics, and adds nonlinear cochlear activity.

There is a the growing body of evidence that the TM is a critical structure in the precise nature of mammalian hearing [34], especially frequency filtering. Working with the TM in isolation can provide valuable insight into its mechanical properties, though we emphasize that the results we get with this model require the components be assembled as a system. We have demonstrated that TM properties affect a transition in the phase of the hair bundle shear that limits the effectiveness of OHCs in amplifying BM vibrations at low frequencies, increasing selectivity.

## Supporting Information

File S1
**Figure S1, Frequency responses due to change in TM damping.** More TM damping leads to a smoother curve, but does not significantly shift the peak or the frequencies of the phase transition. Sensitivity increases with selectivity. **Figure S2, Frequency responses due to change in OC stiffness.** In this case, increased coupling between the TM and BM leads to a sharper, bumpier curve, and sensitivity increases with selectivity. **Figure S3, Frequency responses due to change in BM stiffness.** Changing the BM stiffness leads to a trade in sensitivity for selectivity. In this case, we can observe that the peak frequency increases with BM stiffness, as expected, while the phase difference remains the same. This is compatible with our band pass filter description of the tuning mechanism, but in this case we are moving the high frequency corner. **Figure S4, Diagram of the three masses of the cochlear partition and the forces on them per unit length.** Arrows are intended to depict direction of force due to positive displacements. The direction of 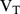 depends on TM position. **Table S1, Viscoelastic Parameters. Table S2, Structural Dimensions.**
(PDF)
