# Supplementary material for: Phase of Shear Vibrations within Cochlear Partition Leads to Activation of the Cochlear Amplifier
Source: PLoS One. 2014 Feb 14;9(2):e85969. doi: 10.1371/journal.pone.0085969 (PMC3925081; doi:10.1371/journal.pone.0085969)
Supplement: File S1 — Figure S1, Frequency responses due to change in TM damping. More TM damping leads to a smoother curve, but does not significantly shift the peak or the frequencies of the phase transition. Sensitivity increases with selectivity. Figure S2, Frequency responses due to change in OC stiffness. In this case, increased coupling between the TM and BM leads to a sharper, bumpier curve, and sensitivity increases with selectivity. Figure S3, Frequency responses due to change in BM stiffness. Changing the BM stiffness leads to a trade in sensitivity for selectivity. In this case, we can observe that the peak frequency increases with BM stiffness, as expected, while the phase difference remains the same. This is compatible with our band pass filter description of the tuning mechanism, but in this case we are moving the high frequency corner. Figure S4, Diagram of the three masses of the cochlear partition and the forces on them per unit length. Arrows are intended to depict direction of force due to positive displacements. The direction of depends on TM position. Table S1, Viscoelastic Parameters. Table S2, Structural Dimensions. (PDF) [file pone.0085969.s001.pdf]

Numerical Parameters

Structure	Stiffness Coefficient $\times e^{-1\text{mm}^{-1}z}$ N/mm^2	Damping Coefficient $\times 10^{-7} e^{-0.5\text{mm}^{-1}z}$ N-s/mm^2
BM	0.71	0.7
TM	0.43	5.7
OC	0.11	1.4
Gap	0.003	1.4

Table S1: Viscoelastic Parameters

Heights (μm)		Other
BM	23	$L = 7$ mm
TM	30	$W(z) = 1e^{0.1\text{mm}^{-1}z}$ mm
RL	23	$\theta = 10^\circ$
SV & ST	350	$l_s = 0.1$ nm
		$F_A = 2.8 \times 10^{-8}$ N/mm

Table S2: Structural Dimensions

Additional figures

The tables associated with the figures in this section demonstrate selectivity (Q) and sensitivity (maximum velocity) changes with varying model parameters. Top figures show velocity and bottom the phase difference between shear and BM vibrations. All calculations are made at $z = 1.4$ mm and damping and stiffness are given in cgs units per length dynes/cm^2 and dynes-s/cm^2.

	D_{TM}	Q	Max Vel
	1.4	4.8	2.4×10^{-5}
	2.8	4.2	1.7×10^{-5}
	5.7	2.9	9.3×10^{-6}

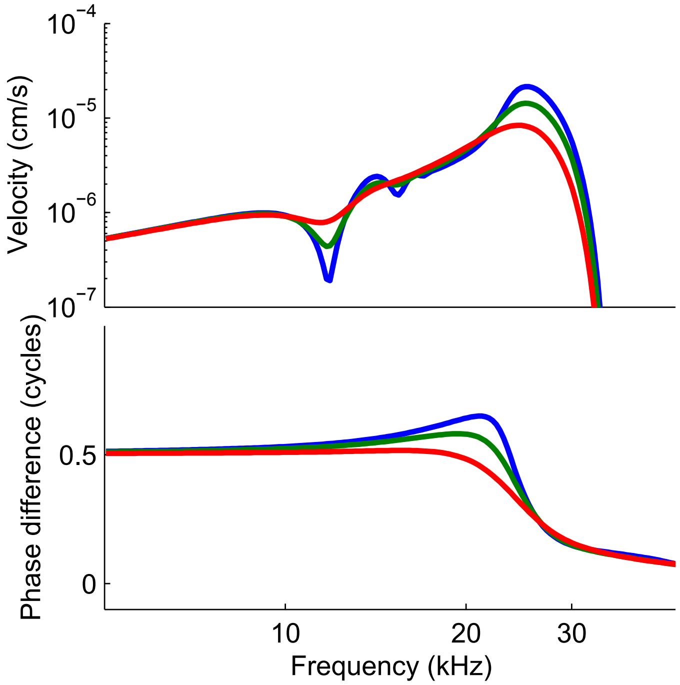

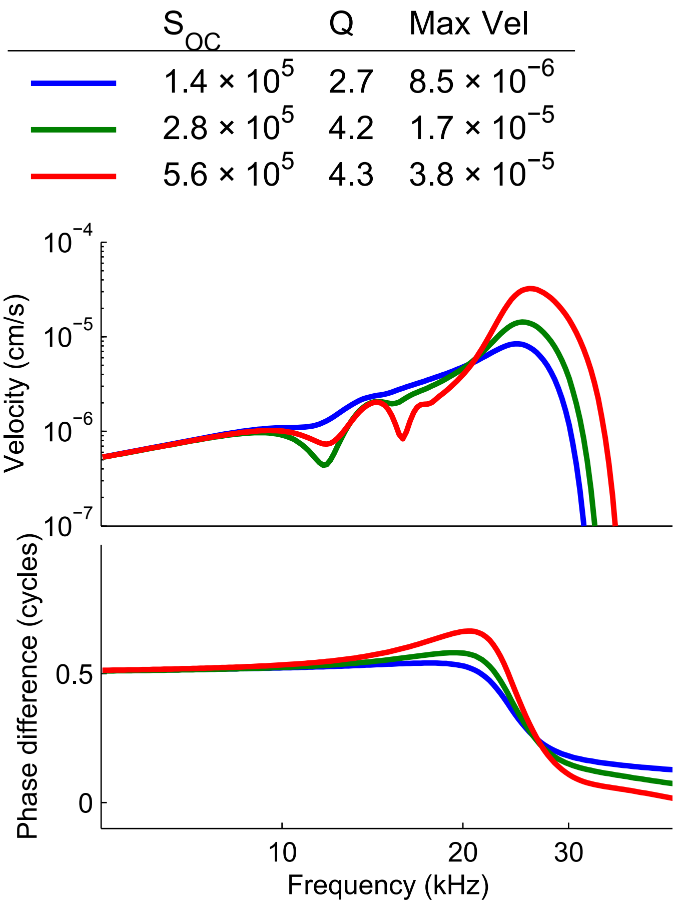
S_OC
Q
Max Vel
1.4 × 10^5
2.7
8.5 × 10^−6
2.8 × 10^5
4.2
1.7 × 10^−5
5.6 × 10^5
4.3
3.8 × 10^−5
10^−4
10^−5
10^−6
10^−7
Velocity (cm/s)
Phase difference (cycles)
0.5
0
10
20
30
Frequency (kHz)

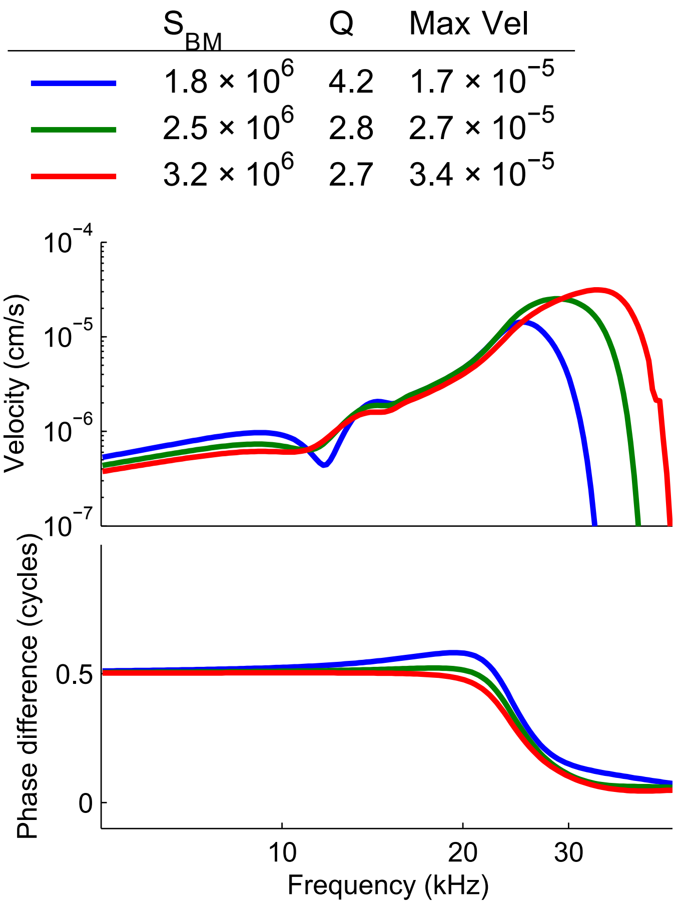
S_BM Q Max Vel
1.8 × 10^6 4.2 1.7 × 10^−5
2.5 × 10^6 2.8 2.7 × 10^−5
3.2 × 10^6 2.7 3.4 × 10^−5
Velocity (cm/s)
10^−4
10^−5
10^−6
10^−7
Phase difference (cycles)
0.5
0
10
20
30
Frequency (kHz)

Model derivation

Introduction

This supplement gives a detailed derivation of the three degree of freedom model employed in the paper "Phase of shear vibrations within the cochlear partition leads to activation of the cochlear amplifier." It is intended to integrate previous work with new material, and to present details of the mathematical derivation for the ease of the reader. Because it is intended to be complimentary to the main text, we will avoid reiteration of definitions and subscripts presented there. There is one departure from the notation in the main text; here we use double subscripts to enumerate a WKB expansion and active force expansion, but in the main text discussion of the WKB expansion is extremely abbreviated, and for clarity we only enumerated the active force expansion.

The goal is to find a solution to the general partition equation

$$\Gamma\vec{A}(z,t) = W(z)\vec{P}(x,0,z,t) + \vec{F}_{\mathrm{A}} \tag{S1}$$

given our specific model of the cross section and cochlear activity. It describes the interaction of the viscoelastic partition elements (Γ), the partition displacement ($\vec{A}$), the fluid pressure ($\vec{P}$) and the active force ($\vec{F}_{\mathrm{A}}$). We will solve Laplace's equation in a long, narrow tube with a single vibrating boundary by employing a WKB expansion based on the asymmetry of the tube. This will allow us to express the fluid pressure analytically in terms of the partition displacement. Using a force-balance approach we then analyze the interaction of the three masses in the partition, deriving a partition matrix.

At this point, we will be able to write the partition equation such that most terms are linear in $\vec{A}$. The active force is small compared to the inertial forces, we will enable an additional expansion. This gives a homogeneous expression equivalent to a passive model of the cochlea, and the active model will finally be solved in sequence. To summarize, first we will derive an expression for the fluid part of the model, then the partition part. Then we put them together and solve the passive equation, and finally we calculate the active contribution as a perturbation.

Fluid pressure with a single vibrating boundary: Laplace's equation using WKB

The fluid problem is one of multiple scales that we will exploit with the WKB expansion. To separate these scales, it is useful to express the dimensional coordinates (depicted in Fig. 1 of the main text) as the normalized, dimensionless quantities $x^* = x/W(0), y^* = y/W(0)$ and $z^* = z/L$ where $W(0)$ and L, represent short and long dimensions of the cochlea. We also normalize the wavenumber $k^*(z) = W(0)k(z)$.

These normalizations give rise to the small expansion parameter $\varepsilon = W(0)/L$. P, A and Φ take the form $A(\vec{z},\omega) = (\vec{A}_0(z^*,\omega) + \varepsilon\vec{A}_1(z^*,\omega) + \ldots) = (\vec{A}_0(z^*) + \varepsilon\vec{A}_1(z^*) + \ldots)e^{i\phi^*(k^*(z^*),\omega)}$ where $\phi^*(k^*(z^*),\omega) = \omega t - 1/\varepsilon \int_0^{z^*} k^*(\eta)\,\mathrm{d}\eta$.

To solve for the pressure we start by writing Laplace's equation for Φ

$$0 = \nabla^2\Phi(x,y,z,t). \tag{S2}$$

Substituting the normalizations from above and multiplying by $W^2(0)$, using $_\mathrm{T}$ to indicate derivatives in the transverse dimensions x^* and y^*, we have

$$\begin{aligned}
0 &= \left(\nabla_\mathrm{T}^2 + \varepsilon^2\frac{\partial^2}{\partial z^{*2}}\right)\left(\Phi_0(x^*,y^*,z^*)e^{i\phi^*(k^*(z^*),\omega)} + \varepsilon\Phi_1(x^*,y^*,z^*)e^{i\phi^*(k^*(z^*),\omega)}\right) + \ldots \\
&= [\nabla_\mathrm{T}^2\Phi_0(x^*,y^*,z^*) + \varepsilon\nabla_\mathrm{T}^2\Phi_1(x^*,y^*,z^*) \\
&\quad -k^{*2}(z^*)\Phi_0(x^*,y^*,z^*) + \varepsilon k^{*2}(z^*)\Phi_1(x^*,y^*,z^*) \\
&\quad -\varepsilon\left(2ik^*\Phi_0'(x^*,y^*,z^*) + ik^{*\prime}(z^*)\Phi_0(x^*,y^*,z^*)\right)]
\end{aligned}$$

where $'$ denotes a derivative in $z*$. Thus the first two equations of the expansion are

$$\mathrm{O}(1) \;\Rightarrow\; \nabla_\mathrm{T}^2\Phi_0(x^*,y^*,z^*) - k^{*2}(z^*)\Phi_0(x^*,y^*,z^*) = 0 \tag{S3}$$

$$\begin{aligned}
\mathrm{O}(\varepsilon) \;\Rightarrow\; &\nabla_\mathrm{T}^2\Phi_1(x^*,y^*,z^*) - k^{*2}(z^*)\Phi_1(x^*,y^*,z^*) \\
&= i\left(k^*(z)\Phi_0^2(x^*,y^*,z^*)\right)'\Phi_0^{-1}(x^*,y^*,z^*)
\end{aligned} \tag{S4}$$

We consider Neumann boundary conditions in the lower chamber, where vibrating boundary is at $y^* = 0$. The fluid velocity in y^* must be equal to the velocity of the partition, thus the derivative of the potential field is related to the

partition displacement by $\frac{\partial \Phi_0(x^*,y^*,z^*,t)}{\partial y^*}|_{y^*=0} = \omega W(0) A_{B0}(z^*,t)$ for $O(1)$ and similar for $O(\varepsilon)$. The other walls are hard, meaning $\frac{\partial \Phi(x^*,y^*,z^*,t)}{\partial n_T} = 0$, and since the height of the chamber is constant, $\frac{\partial \Phi(x^*,y^*,z^*,t)}{\partial y^*}|_{y^*=-H/W(0)} = 0$ exactly. However, the tapered sidewalls are not quite perpendicular to x^*. To find the boundary conditions we must explicitly define the normal derivative for the wall function $\mathscr{F} = 1/(W(0))(x^* - .5W(z^*))$. These boundary conditions are

$$\begin{aligned}\frac{\partial \Phi_0(x^*,y^*,z^*,t)}{\partial x^*} &= 0\\ \frac{\partial \Phi_1(x^*,y^*,z^*,t)}{\partial x^*} &= \mp ik^*(z^*)\frac{W'(z^*)}{2W(0)}\Phi_0(x^*,y^*,z^*,t)\end{aligned}$$

at $x* = \pm\frac{W(z)}{2W(0)}$.

While the normalized coordinates are extremely useful for deriving these equations, it can be tedious to continue with them for the entire calculation. We return to regular coordinates by multiplying Eq. S4 and Eq. S3 by $1/W^2(0)$, the boundary conditions by $1/W(0)$, and noting that $1/\varepsilon \int_0^{z^*} k^*(\eta)\,\mathrm{d}\eta = \int_0^z k(\eta)\,\mathrm{d}\eta$.

To solve the unnormalized Eq. S3 in the lower fluid compartment we see that no x dependence is needed to meet the boundary conditions. We then recognize that the - in front of the k^2 term instructs us to look for hyperbolic solutions in y, and to get 0 on the boundary at $-H$ we need a solution of the form $\Phi_0(y,z) = C\cosh(k(z)(y+H))$. Applying boundary condition at $y=0$ and solving for C gives us

$$\Phi_0(y,z,t) = i\omega\frac{\cosh[k(z)(y+H)]}{k(z)\sinh Hk(z)}A_{B0}(z,t). \tag{S5}$$

To find $\Phi_1(0,z)$ in terms of $A_0(z)$ and $A_1(z)$, use the $O(\varepsilon)$ equations with Green's formula on the cross section of the cochlea.

$$\begin{aligned}&\iint_A (\Phi_0(y,z)\nabla_T^2\Phi_1(y,z) - \Phi_1(y,z)\nabla_T^2\Phi_0(y,z))\,\mathrm{d}A\\ &= \oint \left(\Phi_0(y,z)\frac{\partial \Phi_1(y,z)}{\partial n_T} - \Phi_1(y,z)\frac{\partial \Phi_0(y,z)}{\partial n_T}\right)\mathrm{d}S\end{aligned} \tag{S6}$$

The integral over x on the LHS simply multiplies the expression by $W(z)$. Substitute Eq. S3 and Eq. S4 for terms on the LHS. On the RHS we make use of

the boundary conditions in to write down the terms, and then use Leibniz's rule to combine them.

$$
\begin{aligned}
i\frac{LW(z)}{W(0)}\int_{-H}^{0}(k(z)\Phi_0^2(y,z))'\,\mathrm{d}y &= -i\omega W(z)\Phi_0(0,z)A_{\mathrm{B}1}(z) \\
&\quad -i\frac{LW'(z)}{W(0)}\int_{-H}^{0}k(z)\Phi_0^2(y,z)\,\mathrm{d}y \\
&\quad +i\omega W(z)\Phi_1(0,z)A_{\mathrm{B}0}(z) \\
\left[\frac{LW(z)}{W(0)}\int_{-H}^{0}(k(z)\Phi_0^2(y,z))\,\mathrm{d}y\right]' &= -\omega W(z)\Phi_0(0,z)A_{\mathrm{B}1}(z) \\
&\quad +\omega W(z)\Phi_1(0,z)A_{\mathrm{B}0}(z)
\end{aligned}
$$

Finally we arrive at

$$
\Phi_1(0,z)=\frac{L}{\omega W(0)W(z)A_{\mathrm{B}0}(z)}\left[W(z)\int_{-H}^{0}(k(z)\Phi_0^2(y,z))\,\mathrm{d}y\right]'+\Phi_0(0,z)\frac{A_{\mathrm{B}1}(z)}{A_{\mathrm{B}0}(z)}
$$

The integral evaluates to

$$
\int_{-H(z)}^{0}(k(z)\Phi_{\mathrm{B}0}^2(y,z))\,\mathrm{d}y=-\omega^2\frac{2Hk(z)+\sinh(2Hk(z))}{4k^2(z)\sinh^2(Hk)}A_{\mathrm{B}0}^2(y,z). \tag{S7}
$$

and for compactness we write

$$
G(z)=W(z)\frac{2Hk(z)+\sinh(2Hk(z))}{4k^2(z)\sinh^2(Hk(z))}. \tag{S8}
$$

The well known relation $P=-\rho\partial_t\Phi$ then gives an expression for P proportional to ω^2. Thus the pressure force can be thought of as the effective fluid mass, m_f, defined in Eq. 5 in the main text.

Using Eq. S5 we can now express P as

$$
P_{\mathrm{T}0}(0,z) = \frac{\omega^2 m_f A_{\mathrm{B}0}(z)}{W(z)} \tag{S9}
$$

$$
P_{\mathrm{T}1}(0,z) = \frac{\omega^2 m_f A_{\mathrm{B}1}(z)}{W(z)}+\frac{-i\rho L}{W(0)W(z)A_{\mathrm{B}0}(z)}\left[W(z)G(z)A_{\mathrm{B}0}^2(z)\right]'. \tag{S10}
$$

The expressions for the pressure in the SV can be similarly derived, and is different from this by a sign change and replacing $A_{\mathrm{B}0}$ with $A_{\mathrm{S}0}\sin\theta+A_{\mathrm{R}0}$.

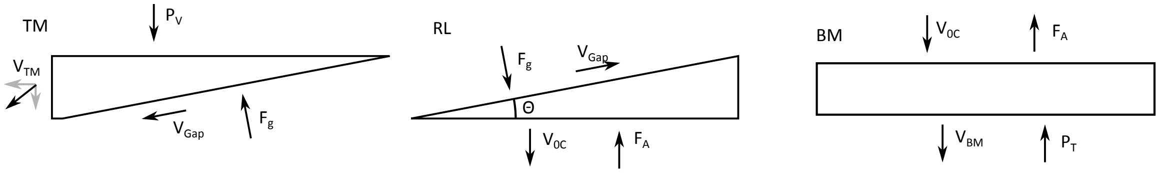

Partition Force Balance

To generate the partition matrix representing the internal mass, damping and elasticity of the cochlear partition, we begin by balancing the forces shown in section . A number of assumptions are involved in writing down the force balance equations as follows.

Time dependence is of the form $e^{i\omega t}$, and thus the forces due to mass acceleration and damping are the $-\omega^2$ and $i\omega$ times the displacement, respectively, so the viscoelastic terms are $V_x = i\omega D_x + S_x$ where x is a subscript defined in Fig. 1 in the main text. The gap width between the RL and TM is constant due to thin film adhesion. To account for this we depict a force, F_g, which is applied equal and opposite on the TM and RL normal to the surface. F_A is the active force. The fluid pressure acts entirely in the y direction on the TM and BM. Subscripts for the pressure (P_x) are V and T for SV and ST, respectively.

Since the TM is the only mass that has any net motion in the x direction, we write four force balance equations, one for the TM in the x direction and the other three for the TM, RL, and BM in the y direction.

$$
\begin{aligned}
-\omega^2 M_{\mathrm{T}} A_{\mathrm{S}} \cos\theta &= -F_g \sin\theta - V_{\mathrm{TM}} A_{\mathrm{S}} \cos\theta - V_{\mathrm{Gap}} A_{\mathrm{S}} \cos\theta && \text{(S11)} \\
-\omega^2 M_{\mathrm{T}} (A_{\mathrm{S}} \sin\theta + A_{\mathrm{T}}) &= -W(z) P_{\mathrm{V}}(0,z) + F_g \cos\theta \\
&\quad - V_{\mathrm{TM}} (A_{\mathrm{S}} \sin\theta + A_{\mathrm{T}}) - V_{\mathrm{Gap}} A_{\mathrm{S}} \sin\theta && \text{(S12)} \\
-\omega^2 M_{\mathrm{R}} A_{\mathrm{T}} &= -F_g \cos\theta + V_{\mathrm{Gap}} A_{\mathrm{S}} \sin\theta \\
&\quad - V_{\mathrm{OC}} (A_{\mathrm{T}} - A_{\mathrm{B}}) - F_A && \text{(S13)} \\
-\omega^2 M_{\mathrm{B}} A_{\mathrm{B}} &= W(z) P_{\mathrm{T}}(0,z) + V_{\mathrm{OC}} (A_{\mathrm{T}} - A_{\mathrm{B}}) \\
&\quad - V_{\mathrm{BM}} A_{\mathrm{B}} + F_A && \text{(S14)}
\end{aligned}
$$

These equation are not linearly independent. We can reduce by one equation and one unknown by solving Eq. S12 for F_g and substituting into Eq. S11. Grouping the passive, internal partition forces on one side of the equation we have

$$-\omega^2 M_{\mathrm{T}}(A_{\mathrm{S}}+A_{\mathrm{T}}\sin\theta)+(V_{\mathrm{T}}+V_{\mathrm{G}})A_{\mathrm{S}}+V_{\mathrm{T}}A_{\mathrm{T}}\sin\theta=-W(z)P_{\mathrm{V}}(0,z)\sin\theta. \tag{S15}$$

Also by eliminating F_g in Eq. S13 we have

$$-\omega^2 M_{\mathrm{T}}A_{\mathrm{S}}\sin\theta-\omega^2(M_R+M_{\mathrm{T}})A_{\mathrm{T}}+V_{\mathrm{T}}(A_{\mathrm{S}}\sin\theta+A_{\mathrm{T}})-V_{\mathrm{O}}(A_{\mathrm{T}}-A_{\mathrm{B}})$$
$$=-W(z)P_{\mathrm{V}}(0,z)-F_A. \tag{S16}$$

Equations (S14-S16) lead to Eqs. (2) and (3) and the definition of the fluid coefficient matrix $\mathbf{M}_f$ in the main text.

Active Force Expansion

In the previous section we derived the matrix for $\mathbf{\Gamma}=-\omega^2\mathbf{M}+\mathbf{V}$ We also have expressions for P in both compartments, and Eqs. (S14 - S16) give us the appropriate vector representation. Using the first order WKB terms, rewrite Eq. S1 as

$$(\mathbf{\Gamma}-\omega^2 m_f\mathbf{M}_f)\vec{A}_0(z,t)=\vec{F}_A \tag{S17}$$

where the active force $\vec{F}_{\mathrm{A}}=f_{\mathrm{A}}\Psi(A_{\mathrm{S}00}(z))[0,-1,1]^{\mathrm{T}}$ is described in the main text.

As with the previous expansion, we want to emphasize the different scales of the problem through normalization. To do so we define $\mathbf{\Gamma}^*=\mathbf{\Gamma}/(\omega^2 M_{\mathrm{B}}(0))$ and $m_f^*=m_f/M_{\mathrm{B}}(0)$. We also write $\vec{A}_0^*(z,t)=\vec{A}_0(z,t)/l_s$ where l_s is a characteristic saturation length defined by $.99=\psi(l_s)$. The expansion term $\delta=f_a/(\omega^2 M_{\mathrm{B}}(0)l_s)$ is created by dividing by the denominator. We expand $\vec{A}(z,t)$ and $k(z,t)$ as

$$\vec{A}^*{}_0(z,t) = \vec{A}^*{}_{00}(z,t)+\delta\vec{A}^*{}_{01}(z,t)\ldots \tag{S18}$$
$$k = k_0+\delta k_1\ldots \tag{S19}$$

In order to identify the O(1) equation, we need to use the expansions

$$\begin{aligned} \frac{1}{k_0+\delta k_1} &= \frac{1}{k_0}-\delta\frac{k_1}{k_0^2}+\dots \\ \coth(k_0+\delta k_1) &= \coth k_0+\delta(k_1-k_1\coth^2 k_0) \end{aligned}$$

We replace $k(z)$ with $k_0(z)$ in m_f and introduce the expansion coefficient

$$p_f=\frac{1}{\coth(Hk_0)}-\coth(Hk_0)-\frac{1}{Hk_0}$$

The O(1) and O(δ) equations are therefore

$$\begin{aligned} \left(\boldsymbol{\Gamma}^*-m_f^*\mathbf{M_f}\right)\vec{A^*}_{00}(z) &= 0 &\text{(S20)} \\ \left(\boldsymbol{\Gamma}^*-m_f^*\mathbf{M}_f\right)\vec{A^*}_{01}(z) &= m_f^* p_f H k_1 \mathbf{M}_f\vec{A}_{00}^*(z) \\ &\quad +\Psi(A_{S00}(z)) &\text{(S21)} \end{aligned}$$

The unnormalized versions are Eqs. 9 and 10 in the main text.

Passive Solutions

To find k_0 we multiply Eq. S20 by $\omega^2 M_{\mathrm{B}}(0)l_s$ and write the eigenvalue problem

$$\boldsymbol{\Gamma}\vec{A}_{00}(z,t)=\omega^2 m_f\mathbf{M}_f\vec{A}_{00}(z,t) \tag{S22}$$

We solve this problem computationally to find the eigenvalues m_f and eigenvectors. As discussed in the main body of the paper, there are two solutions. Most of the solution is can be worked for each mode independently, so we employ distinguishing superscripts only for equations that involve both. The expression for m_f gives an infinite number of values for k_0, but only one wavenumber will meet the requirements of a forward propagating wave (positive real part and any imaginary part being negative and of much smaller absolute value.)

We can write each eigenvector using the notation

$$\vec{A}_{00}(z)=\gamma_0(z)\begin{pmatrix}\alpha(z)\\ \beta(z)\\ 1\end{pmatrix}=\begin{pmatrix}A_{\mathrm{S00}}(z)\\ A_{\mathrm{T00}}(z)\\ A_{\mathrm{B00}}\end{pmatrix} \tag{S23}$$

The solution to the eigenvalue problem provides $\alpha(z)$ and $\beta(z)$, but to find $\gamma_0(z)$ we must consider the higher order expressions of the WKB expansion.

$$[\mathbf{\Gamma}-\omega^2 m_f \mathbf{M}_f]\vec{A_{10}}(z) = i\rho\omega^2 \begin{bmatrix} -\frac{\sin\theta}{A_{S00}(z)\sin\theta+A_{R00}(z)}[G(A_{S00}(z)\sin\theta+A_{R00}(z))^2]' \\ -\frac{1}{A_{S00}(z)\sin\theta+A_{R00}(z)}[G(A_{S00}(z)\sin\theta+A_{R00}(z))^2]' \\ -\frac{1}{A_{B00}(z)}[GA_{B00}^2(z)]' \end{bmatrix}. \quad \text{(S24)}$$

We can see the homogeneous equation is Eq. S22, so the determinant of $(\mathbf{\Gamma}-\omega^2 m_f \mathbf{M}_f)$ must be 0. Such an equation must satisfy specific requirements to be solved. If the RHS of the equation is abbreviated $\vec{F_0}$ the solvability condition is

$$\vec{F_0}\cdot\vec{\eta_0}=0 \quad \text{(S25)}$$

where $\vec{\eta_0}$ is the eigenvector of the adjoint matrix $(\mathbf{\Gamma}-m_f\mathbf{M}_f)^\dagger$. Due to the symmetry of the matrix, $\vec{\eta_0}=\vec{A_{00}}$. Substituting $\vec{A_{00}}$ using the notation in Eq. S23 into Eq. Eq. S25 and integrating we get

$$[(\alpha(z)\sin\theta+\beta(z))^2+1]\gamma^2(z)G(z)=C \quad \text{(S26)}$$

where C is the energy for each mode, which is constant in z.

Once the fluid pressure, is determined at $z=0$ the equations above can be used to fully solve the passive problem. To do this we make use of both modes of the problem. Incompressibility dictates the integral of longitudinal fluid flows, $V(y,z)$ of both compartments is 0 at $z=0$. Furthermore, $V(y,z)=kP(y,z)$. Thus

$$\begin{aligned} 0 &= \int_0^H \left(k^{\mathrm{T}}(z)P_{\mathrm{V}}^{\mathrm{T}}(y,0)+k^{\mathrm{B}}(z)P_{\mathrm{V}}^{\mathrm{B}}(y,0)\right)\mathrm{d}y \\ &\quad \int_{-H}^0 \left(k^{\mathrm{T}}(z)P_{\mathrm{V}}^{\mathrm{T}}(y,0)+k^{\mathrm{B}}(z)P_{\mathrm{V}}^{\mathrm{B}}(y,0)\right)\mathrm{d}y \\ &= \gamma^{\mathrm{T}}(0)\frac{\alpha^{\mathrm{T}}(0)\sin\theta+\beta^{\mathrm{T}}(0)}{k^{\mathrm{T}}(0)}+\gamma^{\mathrm{B}}(0)\frac{\alpha^{\mathrm{B}}(0)\sin\theta+\beta^{\mathrm{B}}(0)}{k^{\mathrm{B}}(0)} \\ &\quad -\gamma^{\mathrm{T}}(0)\frac{1}{k^{\mathrm{T}}(0)}-\gamma^{\mathrm{B}}(0)\frac{1}{k^{\mathrm{B}}(0)} \\ \frac{\gamma^{\mathrm{T}}(0)}{\gamma^{\mathrm{B}}(0)} &= -\frac{k^{\mathrm{T}}(0)}{k^{\mathrm{B}}(0)}\left(\frac{\alpha^{\mathrm{B}}(0)\sin\theta+\beta^{\mathrm{B}}(0)-1}{\alpha^{\mathrm{T}}(0)\sin\theta+\beta^{\mathrm{T}}(0)-1}\right) \end{aligned} \quad \text{(S27)}$$

From this we get the relation between $\gamma^{\mathrm{T}}(0)$ and $\gamma^{\mathrm{B}}(0)$. From here we use the stapes pressure to determine the values.

$$
\begin{aligned}
P_s &= \frac{1}{H}\int_0^H \left(P^{\mathrm{T}}_{\mathrm{V0}}(1)(y,0)+P^{\mathrm{B}}_{\mathrm{V0}}(y,0)\right)\mathrm{d}y \\
&= \frac{-\omega^2\rho}{H}\left(\frac{\alpha^{\mathrm{T}}(0)\sin\theta+\beta^{\mathrm{T}}(0)}{(k^{\mathrm{T}}(0))^2}\gamma_0^{\mathrm{T}}(0)+\frac{(\alpha^{\mathrm{B}}(0)\sin\theta+\beta^{\mathrm{B}}(0))}{(k^{\mathrm{B}}(0))^2}\gamma_0^{\mathrm{B}}(0)\right) \\
&= \frac{-\omega^2\rho}{H}\left[\frac{(\alpha^{\mathrm{T}}(0)\sin\theta+\beta^{\mathrm{T}}(0))}{(k^{\mathrm{T}}(0))^2}\right. \\
&\quad\left.-\frac{(\alpha^{\mathrm{B}}(0)\sin\theta+\beta^{\mathrm{B}}(0))(\alpha^{\mathrm{T}}(0)\sin\theta+\beta^{\mathrm{T}}(0)-1)}{(\alpha^{\mathrm{B}}(0)\sin\theta+\beta^{\mathrm{B}}(0)-1)k^{\mathrm{T}}(0)k^{\mathrm{B}}}\right]\gamma_0^{\mathrm{T}}(0) \\
\gamma_0^{\mathrm{T}}(0) &= \frac{-P_sHk^{\mathrm{T}}(0)(\alpha^{\mathrm{B}}(0)\sin\theta+\beta^{\mathrm{B}}(0)-1)}{\omega^2\rho} \\
&\quad\times\left[\frac{(\alpha^{\mathrm{T}}(0)\sin\theta+\beta^{\mathrm{T}})(\alpha^{\mathrm{B}}(0)\sin\theta+\beta^{\mathrm{B}}(0)-1)}{k^{\mathrm{T}}(0)}\right. \\
&\quad\left.-\frac{(\alpha^{\mathrm{B}}(0)\sin\theta+\beta^{\mathrm{B}})(\alpha^{\mathrm{T}}(0)\sin\theta+\beta^{\mathrm{T}}(0)-1)}{k^{\mathrm{B}}(0)}\right]^{-1} \qquad \text{(S28)}
\end{aligned}
$$

A similar expression with the modes switched gives $\gamma_0^{\mathrm{B}}(0)$.

Perturbed Equation Solutions

Calculating k_1 involves using the same solvability condition we applied above. The orthogonality relation for this problem is

$$
\vec{A_{00}}(z,t)\cdot\left[\omega^2 m_f p_f H k_1 \mathbf{M}_f \vec{A}_{00}(z,t)+\omega^2 M_{\mathrm{B}}(0) l_s \vec{F_A}\right]=0. \qquad \text{(S29)}
$$

Solving for k_1

$$
\begin{aligned}
0 &= m_f p_f H k_1\left[\sin^2\theta A_{\mathrm{S00}}(z,t)+\sin\theta A_{\mathrm{T00}}(z,t)\right]A_{\mathrm{S00}}(z,t) \\
&\quad + m_f p_f H k_1\left[\sin\theta A_{\mathrm{S00}}(z,t)+A_{\mathrm{T00}}(z,t)\right]A_{\mathrm{T00}} - M_{\mathrm{B}}(0) l_s \Psi(z,t) A_{\mathrm{T00}}(z,t) \\
&\quad + m_f p_f H k_1\left[A_{\mathrm{T00}}(z,t)\right]^2 + M_{\mathrm{B}}(0) l_s \Psi(z,t) A_{\mathrm{B00}}(z,t) \\
0 &= m_f p_f H k_1 \gamma_0(z)[\alpha^2(z)\sin^2\theta+2\sin\theta\,\alpha(z)\beta(z)+\beta^2(z)+1] \\
&\quad + M_{\mathrm{B}}(0)\Psi(z) l_s(\beta(z)-1) \\
k_1 &= \frac{M_{\mathrm{B}}(0) l_s \Psi(z)(1-\beta(z))}{m_f p_f H \gamma_0(z)\left[(\alpha(z)\sin\theta+\beta(z))^2+1\right]} \qquad \text{(S30)}
\end{aligned}
$$

We use Eq. S30 to find k_1 separately for each mode by substituting one eigenvalue m_f (and using the propagating value of k_0 to determine p_f. Once the solvability condition has been met, the solution $\vec{A}_{10}(z,t)$ to a problem like this where the determinant of the LHS is equal to 0 is made of the homogeneous solution plus a particular solution. We know the homogeneous solution from the unperturbed problem (though we don't necessarily know how it is normalized).

When the homogeneous problem is an eigenvalue problem such as this, the particular solution to the nonhomogeneous problem will be a linear combination of the eigenvectors NOT corresponding to the eigenvalue currently used. In this case there is only one alternate eigenvector. To determine the appropriate coefficient, which we will denote as γ_1, we need to find another boundary condition or make an assumption. In this case, we assume the homogeneous solution makes 0 contribution to the active all of the action is contained in the passive solution.

Using subscripts to denote the elements of $\boldsymbol{\Gamma}$, using the first equation in the system Eq. S21 and substituting Eq. S30 we have

$$\begin{aligned}
& \gamma_1^{\mathrm{T}}\left[\alpha^{\mathrm{B}}(z)\left(\Gamma_{11}-\omega^2 m_f^{\mathrm{T}}\sin^2\theta\right)+\beta^{\mathrm{B}}(z)\left(\Gamma_{12}-\omega^2 m_f^{\mathrm{T}}\sin\theta\right)+\Gamma_{13}\right] \\
& =\frac{M_{\mathrm{B}}(0) l_s \Psi^{\mathrm{T}}(z)(1-\beta^{\mathrm{T}}(z))}{m_f^{\mathrm{T}} p_f^{\mathrm{T}} H \gamma_0^{\mathrm{T}}(z)\left[(\alpha^{\mathrm{T}}(z)\sin\theta+\beta^{\mathrm{T}}(z))^2+1\right]} \\
& \times m_f^{\mathrm{T}} p_f^{\mathrm{T}} H \gamma_0^{\mathrm{T}}(z)(\alpha^{\mathrm{T}}(z)\sin^2\theta+\beta^{\mathrm{T}}\sin\theta) \\
\gamma_1^{\mathrm{T}}(z) \quad = \quad & \Psi^{\mathrm{T}}(z)\frac{M_{\mathrm{B}}(0) l_s(1-\beta^{\mathrm{T}}(z))(\alpha^{\mathrm{T}}(z)\sin^2\theta+\beta^{\mathrm{T}}(z)\sin\theta)}{(\alpha^{\mathrm{T}}\sin\theta+\beta^{\mathrm{T}})^2+1} \\
& \times[\alpha^{\mathrm{B}}\Gamma_{11}^{\mathrm{T}}+\beta^{\mathrm{B}}\Gamma_{12}^{\mathrm{T}}+\Gamma_{13}^{\mathrm{T}} \\
& -\omega^2 m_f^{\mathrm{T}}(\alpha^{\mathrm{B}}(z)\sin^2\theta+\beta^{\mathrm{B}}(z)\sin\theta)]^{-1} \qquad \text{(S31)}
\end{aligned}$$

Using both modes, we have equations for all the quantities needed to calculate the

full partition displacements

$$\vec{A}(z,t) = \left[\gamma_0^{\mathrm{T}}\begin{pmatrix}\alpha^{\mathrm{T}}\\ \beta^{\mathrm{T}}\\ 1\end{pmatrix} + \delta\gamma_1^{\mathrm{T}}\begin{pmatrix}\alpha^{\mathrm{B}}\\ \beta^{\mathrm{B}}\\ 1\end{pmatrix}\right] e^{i(\omega t - \int_0^z (k_0^{\mathrm{T}}(\eta)+\delta k_1^{\mathrm{T}}(\eta))\,\mathrm{d}\eta} + \left[\gamma_0^{\mathrm{B}}\begin{pmatrix}\alpha^{\mathrm{B}}\\ \beta^{\mathrm{B}}\\ 1\end{pmatrix} + \delta\gamma_1^{\mathrm{B}}\begin{pmatrix}\alpha^{\mathrm{T}}\\ \beta^{\mathrm{T}}\\ 1\end{pmatrix}\right] e^{i(\omega t - \int_0^z (k_0^{\mathrm{B}}(\eta)+\delta k_1^{\mathrm{B}}(\eta))\,\mathrm{d}\eta}. \quad \text{(S32)}$$
